# Prevalence of Chronic Inducible Urticaria in Elderly Patients

**DOI:** 10.3390/jcm10020247

**Published:** 2021-01-12

**Authors:** Maddalena Napolitano, Gabriella Fabbrocini, Luca Stingeni, Cataldo Patruno

**Affiliations:** 1 Department of Medicine and Health Sciences Vincenzo Tiberio, University of Molise, 86100 Campobasso, Italy; 2 Section of Dermatology, Department of Clinical Medicine and Surgery, University of Naples Federico II, 80131 Naples, Italy; gafabbro@unina.it; 3 Dermatology Section, Department of Medicine, University of Perugia, 06129 Perugia, Italy; luca.stingeni@unipg.it; 4 Department of Health Sciences, University Magna Graecia of Catanzaro, 88100 Catanzaro, Italy; cataldo.patruno@unicz.it

**Keywords:** inducible chronic urticaria, physical urticaria, elderly

## Abstract

Background: No data currently exist regarding the epidemiology of chronic inducible urticarias (CIndUs) in the ≥65-year-old population. Objective: The study aimed to determine the prevalence of CIndUs among elderly patients affected by chronic urticaria (CU). Methods: The medical records of all patients referred to us with a diagnosis of CU from January 2008 to September 2020 were retrospectively reviewed, and the patients with CIndUs were identified. The subjects aged 65 years or above were included in the study. Results: The number of patients aged 65 years or above was 153 out of 1970 subjects affected by CU (7.77%; 92 females (60.13%); mean age 70.96 ± 4.22). Out of 153, 26 patients (16.99%; 20 females (76.9%); mean age 71.23 ± 2.6 years) were diagnosed with CIndUs. Most subjects (25/26; 96.15%) suffered from physical urticarias. Symptomatic dermographism was the most frequent, affecting 65.38% (17/26) of our patients, followed by cold urticaria (6/26 (23.08%) cases). Conclusion: Our data seem to indicate that CIndUs may also affect the elderly, although it occurs less frequently in aging patients than in lower age groups.

## 1. Introduction

Chronic urticaria (CU) is characterized by recurrent wheals and/or angioedema lasting for more than six weeks [1]. It is a common disease that significantly affects the quality of life of patients [1,2]. The prevalence of CU in adults is estimated at 0.5% to 5%, while it affects 0.1% to 3% of children [3,4,5].

According to current guidelines, CU can be classified as chronic spontaneous urticaria (CSU) in the absence of an identifiable trigger and as chronic inducible urticarias (CIndUs), when wheal or angioedema development can be triggered through exposure to a specific stimulus [4]. CIndUs can be divided into two groups: physical urticarias (PUs) and nonphysical urticarias (NPUs) (Table 1) [6,7].

PUs are classified according to the physical trigger and include symptomatic dermographism, cold or heat urticarias, delayed pressure urticaria, solar urticaria, and vibratory angioedema. NPUs are triggered by other stimuli. In cholinergic urticaria, wheals appear after a crucial rise in the body’s core temperature through activities like exercise or through passive heating (e.g., hot bath). In the case of contact urticaria, wheals appear after skin contact with chemicals, while aquagenic urticaria is induced by an exposition to water [6]. It is important to note that more than one CIndU can coexist in the same individual.

Usually, the diagnosis is based on anamnesis. However, all CIndUs are reproducible with specific simple challenge tests (CTs) that are therefore used for the diagnosis [6,7].

Currently, some tools are capable of evaluating the threshold value of the stimulus eliciting the wheal or angioedema in some CIndUs. Consequently, these tools are particularly useful not only for the diagnosis but also for the assessment of the disease’s severity and the follow-up of its evolution and effectiveness of treatments.

Systemic symptoms rarely occur in CIndUs; acute respiratory involvement, hypotension, lipothymia, and anaphylaxis are occasionally reported in association with cold, cholinergic, or contact urticaria [8,9,10]. Although very rarely, these systemic symptoms may also be elicited by CTs; hence, CTs must be conducted only in health centers with resuscitation equipment and trained personnel [6,7]. Apart from CTs, other clinical investigations are generally not useful in CIndUs, with the exception of some forms of cold urticaria. Indeed, it is reported that cold-induced urticaria may be secondary to infections or drug intake and sometimes associated with cryoglobulinemia or cryofibrinogenemia [11].

The treatment of CIndUs is similar to that of CSU, including the use of antihistamines, the dosage of which can be quadrupled in case of a lack of therapeutic response. Strong evidence supports the use of omalizumab in the treatment of patients with therapy-refractory CIndU, even if more data from randomized controlled studies are warranted [12].

The aging population phenomenon is increasing worldwide. In 2020, 23.1% of the total population in Italy is estimated to be 65 years of age and above [13] and this is expected to increase to 32.6% by the year 2065 [14]. Several studies are investigating etiopathogenesis, epidemiology, clinical presentation, and clinical feature of CU in elderly people [15,16,17,18]. As a result, a 0.23% prevalence of CU has been reported among elderly patients [15]. However, to the best of our knowledge, there are no data regarding the epidemiology and clinical features of CIndUs in the population ≥65 year-old.

The purpose of this study was to evaluate the frequency and clinical characteristics of CIndUs in a group of elderly patients.

## 2. Methods

The study was performed at the Dermatology Unit of the University of Naples Federico II and was approved by the local Ethics Committee (IRB 116/20). We retrospectively reviewed the medical records of all patients referred to us with a diagnosis of CU from January 2008 to September 2020, and patients with CIndUs were identified. All the patients had been examined, tested, and followed by the same dermatologists (C.P or M.N). Since the study aimed to evaluate the prevalence of CIndUs in elderly patients, only subjects 65 years of age or above at the time of our first visit were included. The protocols used for CTs to identify specific CIndU subtypes were based on current guidelines (Table 2) [6,7]. All patients had been informed about any risk involved with testing, and written informed consent forms for diagnostic work-up and the treatment of personal data were signed by each of them.

During the first six years of the study, the diagnosis of cold or heat urticaria was performed through skin contact with an ice cube or with a glass cylinder filled with warm water, respectively. Afterwards, the TempTest^®^ 4.0 (Emosystems GmbH, Berlin, Germany) device was employed.

CTs were completed under similar environmental conditions. The patients were acclimated to the challenge room temperature for at least 15 min before testing. All the patients refrained from systemic symptomatic treatment prior to testing, if possible [6]. The intake of antihistamines should be stopped at least three days before testing (thus allowing five plasma half-lives of drug elimination), and the intake of corticosteroids should be halted seven days before testing [6].

CTs were performed for the most common triggers, friction (dermographism), cold, heat, and pressure in all patients; the other tests were performed in case of suspicion arising from anamnesis.

GraphPad Prism software (v.4.0; GraphPad Software Inc. La Jolla, CA, USA) was used for all statistical analyses. Mean and standard deviations were reported for descriptive variables. Unpaired Student’s *t*-test was used to calculate statistical differences. *p* < 0.05 was considered statistically significant.

## 3. Results

**Figure 1:**
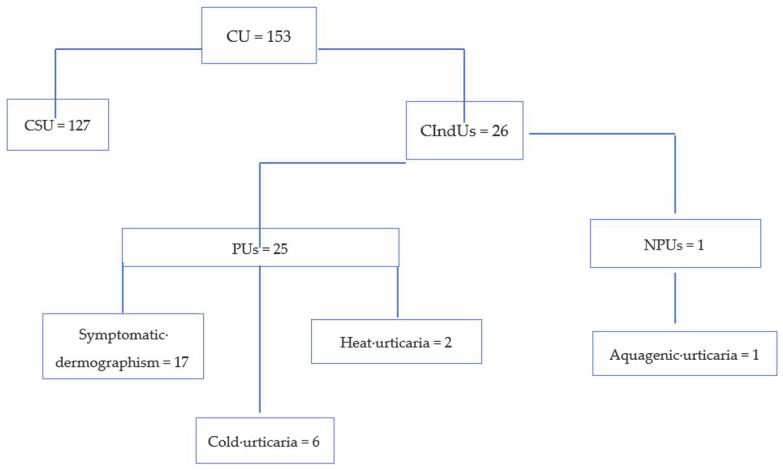
Diagnostic flowchart of elderly patients with chronic urticaria (CU). PU: physical urticarias; NPU: nonphysical urticarias; CIndUs: chronic inducible urticarias; CSU: chronic spontaneous urticaria.

During the study period, a total of 1970 patients (1083 females (54.97%); mean age 47.11 ± 22.8 (range 8–3 years)) affected by CU were referred to our unit. The number of patients 65 years of age or above was 153/1970 (7.77%; 92 females (60.13%); mean age 70.96 ± 4.22), while that of those aged <65 years was 1817/1970 (92.23%; 1071 females (58.94%); mean age 49.26 ± 9.81). Anamnesis and CTs led to the diagnosis of CIndUs in 451/1970 (22.89%) patients, with PUs being diagnosed in 402/451 (89.14%) of the CIndUs patients and NPUs in the other 49/451 (10.42%) subjects. Out of 451, 26 (5.76%) were elderly patients, while 425 (94.24%) patients were <65 year-old. With regard to the individual age groups, the frequency was 26/153 (16.99%) among the elderly (Figure 1) and 425/1817 (23.39%) among younger patients (*p* = 5052). In particular, 25/26 (96.15%) patients of the <65 years group and 377/425 (88.71%) patients of the ≥65 years of age group were affected by PUs (*p* = 0.5952); meanwhile, 1/26 (3.85%) of aging and 48/425 (11.29%) of younger patients suffered from NPUs (*p* = 4250).

In Table 3, the data regarding the group of 26/451 elderly patients with CIndUs were compared to the data of 425/451 CInDUs patients under age of 65 years of age. No significant differences between the elderly and younger CIndUs patients were found for the variables that were considered, probably due to the low number of patients in the elderly group.

As far as the 26 elderly patients are concerned, the mean duration of the disease was 1.9 ± 1.1 years. None of them reported systemic symptoms associated with CIndUs. Due to medical history, no laboratory tests useful for the diagnosis of CIndUs were prescribed to any patient. Most subjects (25/26; 96.15%) suffered from PUs. Symptomatic dermographism was the most frequent type of CIndU, affecting 65.38% (17/26) of our patients (mean value of dermographic tester positive reaction: 3.6 ± 2.4 U.I). Cold urticaria was the second most frequently reported CindUs (6/26 (23.08%) of cases). All the patients with cold urticaria had type I localized urticaria (14). Three patients (50.00%) among them were examined with the ice cube test and three (50.00%) with the TempTest^®^ 4 (mean value 4.5 ± 1.9 °C). Heat contact urticaria was diagnosed in 2/26 (7.69%) patients using the TempTest^®^ 4 (mean value 32 ± 1.41 °C). Delayed pressure urticaria, solar urticaria, and vibratory angioedema were not diagnosed in any patient. NPUs were diagnosed in 1/26 (3.85%) patients who suffered from aquagenic urticaria. No elderly patient was affected by more than one CIndUs. The CTs results were in line with the clinical history of each patient. No serious adverse events were recorded during testing.

Atopy, as current or anamnestic atopic dermatitis, asthma, and/or rhinitis, was found in 12/26 (53.85%). More than one disease coexisted in 5/26 (19.24%) elderly patients compared to 272/425 (64.0%) of subjects <65 years of age [more than one disease coexisted in 81/425 (19.06%)] (Table 4). The difference between the two groups was not significant. Other main comorbidities are reported in Table 4.

All the elderly patients were treated with second-generation antihistamines. The treatment at standard dose was able to significantly reduce the symptoms in 23/26 (88.46%) patients. In 3/26 (11.54%) patients affected by symptomatic dermographism, it was necessary to double the dosage of the antihistamine. No adverse events were reported. These data are similar to those of younger patients. Indeed, all 425 patients below 65 years of age were treated with second-generation antihistamines; the treatment was successful at standard doses in 371 (87.29%; *p* = 0.4834) of them and higher doses in 43 (10.12%; *p* = 0.4250) patients; additionally, 11 (5.99%) unresponsive subjects were treated with off-label drugs, namely cyclosporine in nine (2.12%) and omalizumab in two (0.47%) of them.

## 4. Discussion

Several studies showed that there was no difference in the prevalence of CSU between geriatric and adult patients [15]. However, to the best of our knowledge, there are no current data about the prevalence of CIndUs in patients ≥65 years of age. In this study, the prevalence of CIndUs was 16.99% in a group of 153 CU elderly patients. This prevalence, although not significant, is lower than 23.39% in our patients aged below 65 year-old and lower than 27.1% in a group of 32 out of 118 children younger than 14 years, as reported in our previous experience [19]. The large majority (96.15%) of CIndUs found in the group of elderly individuals were PUs, namely symptomatic dermographism (65.38%), cold urticaria (23.08%), and heat urticaria (7.69%). Only one case (3.85%) of NPU was found, in a patient with aquagenic urticaria. This data seem to confirm that NPUs are more common in young people [20]. However, we did not find significant differences between the two groups of patients by comparing the data related to the individual CIndU, probably due to the small number of elderly patients. Additionally, cases of coexistence of different CIndUs in the same patient were not found in our population of elderly patients but were found in 41/425 (9.65%) younger patients. No differences were found between the two age groups regarding the personal history of atopic diseases. Nevertheless, there were significant differences regarding some comorbidities typical of older age (i.e., arterial hypertension or benign prostatic hyperplasia), which is therefore probably attributable to the different average age of the two groups (71.26 ± 2.61 vs. 44.6 ± 20.9 years).

Generally, there may be some conflicts between the self-reported symptoms of certain CIndUs and specific positive CT results [21]. However, we did not find any discrepancy between the self-reported data and the CT results in our elderly patients. No serious adverse events were recorded during testing, which illustrates the safety of the provocation tests used to identify CIndUs, according to Komarow et al. [21]. Nevertheless, it is appropriate to reiterate that CTs should be conducted in safe conditions for the eventuality, albeit very rare, of side effects. In this case, certain precautions must be taken in the elderly. In particular, testing for cholinergic urticaria should be avoided if there is a history of heart diseases. Therefore, a careful medical history must always be collected and some tests must be performed only when required and in appropriately equipped healthy centers.

Older age is associated with structural and physiological skin changes [15]. A reduction in dermal thickness, a decrease in cutaneous vascularity and cellularity, and reduced histamine skin reactivity are observed with aging [18,21]. Moreover, immune function is remodeled in older age (immunosenescence) [22]. Indeed, the senescence of immune cells leads to a decline of the total and naive B-cells, and of the efficacy of antibody response) [22], thus significantly interfering with the hyper-reactive response commonly found in urticaria. Since the major characteristic of CU pathogenesis is mast cell degranulation, which results in the release of histamine and other chemical mediators [23], it could be speculated that the reduced prevalence of CIndUs in the elderly might be related to a lower number and activity of mast cells. This reduced mast cell reactivity might lead to a reduced ability to respond to trigger factors. Furthermore, some studies on the CU in the elderly reported a smaller number of wheals and a lower prevalence of angioedema in these patients [18,23]. It is possible that among some elderly subjects suffering from severe itch [24], CIndUs (especially dermographism) might be underdiagnosed due to the belief that itching is a common occurrence in the elderly and due to the fact that wheals are not always evident at the time of visit. Hence, the physician should pay particular attention to every case of itching in the elderly and should even suspect the possibility of CIndUs.

Treatment with antihistamines was found to be effective in significantly reducing the symptoms associated with CIndU and to be safe in the elderly. It was necessary to double the dosage in only a minority of cases, as reported by the current recommendations for the patients who are unresponsive to the standard dosage [6,7]. In our experience, the response to the treatment in the elderly was comparable to that of younger patients. These drugs can be safely used and updosed due to low cardiotoxicity. Especially in aging patients, it is necessary to accurately evaluate health conditions in general and heart conditions in particular, and to examine the possible interactions with other drugs [25]. However, no adverse events were reported in our patients.

## 5. Conclusions

This study has limitations that are mainly related to its retrospective nature. First, recording bias is an important possible limitation. Second, the role of aging associated with the modifications of the immune system in the pathophysiology of elderly patients with CIndUs was not investigated. Furthermore, the small number of elderly patients included in this study probably was not enough to give any significant information about the differences with younger groups of patients.

In conclusion, our data seem to indicate that CIndUs, especially PUs, are not uncommon occurrences in the elderly, although supposedly less frequent than in lower age groups. Therefore, in case of symptoms compatible with urticaria and/or angioedema, the clinical history of the patient should be enhanced and CTs—which, in our experience, have proved to be very useful for the diagnosis due to them being simple to perform and safe—should be conducted.

## Figures and Tables

**Table 1:** Classification of chronic urticaria.

*Chronic Urticaria*		
*Chronic Spontaneous Urticaria*	*Chronic Inducible Urticarias*	
	Physical Urticarias	Nonphysical Urticarias
	Symptomatic dermographism Cold urticaria Heat urticaria Delayed pressure urticaria Vibratory angioedema Solar urticaria	Aquagenic urticaria Cholinergic urticaria Contact urticaria

**Table 2:** Main challenge tests used for the diagnosis of CIndUs.

**Physical Urticarias**			
	**Test Site**	**Test**	**Reading Time**
Symptomatic dermographism	Upper Back	Stroking the skin with a pen-sized tool with a spring-loaded blunt tip (dermographic tester *) or with a rectangular plastic tool with four smooth plastic tips of varying lengths (FricTest^®^ 4.0 **)	10 min
Cold urticaria	Volar surface of the forearm	Contact for 5 min with ice cube in a thin plastic bag or thermoelectric Peltier device (TempTest^®^ 4.0 ***)	10 min
Heat urticaria	Volar surface of the forearm	Contact for 5 min with a cylindrical container filled with hot water or TempTest^®^ 4.0 ***	10 min
Delayed pressure urticaria	Shoulder, upper back, thighs, or forearm volar surface	Application for 10 min of a sustained pressure stimulus of 7 kg	6–8 h
Vibratory urticaria	Forearm volar surface	Contact with a flat surface placed on a laboratory vortex at a speed between 780 and 1380 rpm (average, 1000 rpm) for 5 min	10 min
Solar urticaria	Buttocks	Irradiation for 15 min with a projector of UVA (6 J/cm^2^), UVB (60 mJ/cm^2^), and visible light	10 min
**Non-Physical Urticarias**			
	**Test Site**	**Test**	**Reading Time**
Aquagenic urticaria	Side surface of the neck, upper part of the back	Application of a water-drenched compress at body temperature for 20 min	10 min
Cholinergic urticaria		Use of a stationary bike or treadmill until sweating or until the appearance of skin symptoms (not more than 15 min)	During testing, immediately, and 10 min following the end of the test
Contact urticaria	Volar surface of the forearm	Contact with exogenous agents; in negative cases, patch test or prick test. All the tests last 20 min. Determination of a specific IgE level is sometimes needed.	20 min

* HTZ Limited, New Addington, UK. ** Moxie, Berlin, Germany. *** Emosystems GmbH, Berlin, Germany.

**Table 3:** Main features of patients with CIndUs ≥65 years (*n* = 26) and <65 years of age (*n* = 425).

	Patients ≥ 65 Years-Old (*n* = 26)	Patients < 65 Years-Old (*n* = 425)	*p* < 0.05
**Sex**	***n (%)***	***n (%)***	
Males (M)	M 6 (23.08)	M 194 (45.65)	*ns*
Females (F)	F 20 (76.92)	F 231 (54.35)	*ns*
Mean age ± DS (years)	71.26 ± 2.61	46.6 ± 8.61	-
Mean duration of the disease (years)	1.9 ± 1.1	3.7 ± 2.11	*ns*
**Physical Urticarias**	***n (%)***	***n (%)***	
Symptomatic dermographism	17 (65.38)	241 (56.71)	*ns*
Cold urticaria	6 (23.08)	52 (12.24)	*ns*
Heat urticaria	2 (7.69)	15 (3.53)	*ns*
Delayed pressure urticaria	-	12 (2.82)	-
Vibratory angioedema	-	3 (0.71)	-
Solar urticaria	-	13 (3.06)	-
Symptomatic dermographysm + cold urticaria	-	37 (8.71)	-
Symptomatic dermogrtaphyism + heat urticaria	-	4 (0.94)	-
**Non-Physical Urticaria**	***n (%)***	***n (%)***	
Aquagenic urticaria	1/26 (3.85)	16 (3.76)	*ns*
Cholinergic urticaria	-	21 (4.94)	*-*
Contact urticaria	-	11 (2.59)	-

**Table 4:** Comorbidities of patients with CIndUs.

	Patients ≥ 65 Year-Old (*n* = 26) *n (%)*	Patients < 65 Year-Old (*n* = 425) *n (%)*	*p* < 0.05
**Atopic comorbidities**			
Asthma	2 (7.69)	22 (5.18)	*ns*
Rhinitis	9 (34.62)	197 (46.35)	*ns*
Conjunctivitis	1 (3.85)	30 (7.06)	*ns*
Atopic dermatitis	4 (15.38)	92 (21.65)	*ns*
Food allergy	1 (3.85)	12 (2.82)	*ns*
**Other comorbidities**			
Arterial hypertension and cardiovascular disorders	19 (73.01)	101 (23.76)	*ns*
Obesity	5 (19.23)	96 (22.59)	*ns*
Thyroid disease	4 (15.38)	78 (18.35)	*ns*
Benign prostatic hyperplasia	3 (11.54)	15 (3.53)	*ns*
Chronic obstructive pulmonary disease	1 (3.85)	5 (1.18)	
Psychiatric/psychological disorders	2 (7.69)	56 (13.18)	*ns*

## Data Availability

Data is contained within the article.
